# Variation in food web reliance on green and brown energy pathways across ecosystem gradients

**DOI:** 10.1371/journal.pone.0336521

**Published:** 2026-02-04

**Authors:** James W. Sturges, W. Ryan James, Ryan J. Rezek, Rolando O. Santos, Mack White, Gina A. Badlowski, Shakira Trabelsi, Jordan Massie, Justin S. Lesser, Joel C. Trexler, James Nelson, Jennifer S. Rehage

**Affiliations:** 1 Department of Earth and Environment, Institute of Environment, Florida International University, Miami, Florida, United States of America; 2 Department of Biological Sciences, Institute of Environment, Florida International University, Miami, Florida, United States of America; 3 Department of Marine Science, Coastal Carolina University, Conway, South Carolina, United States of America; 4 Department of Biological Science, Florida State University, Florida, United States of America; 5 Department of Marine Sciences, University of Georgia, Athens, Georgia; Gujarat Institute of Desert Ecology, INDIA

## Abstract

Aquatic food webs typically include highly coupled fast, ‘green’ energy pathways driven by algae or phytoplankton and slower, ‘brown’ energy channels driven by detritus and terrestrial plants. Quantifying how much energy biological communities obtain from each of these pathways is essential, particularly across multiple interconnected food webs over large areas, because energy dynamics are known to influence ecosystem structure and function. Despite their importance, few studies track variance in energy channel contributions to food webs across interconnected habitats during distinct hydrologic seasons. In this study, we used tri-isotope Bayesian mixing models to quantify seasonal contributions of energy pathways to consumers in nine aquatic food webs across two river drainages in the Florida coastal Everglades. Sites span an ecosystem gradient from freshwater marshes to estuarine riverine mangroves and marine seagrass habitats. We found that green energy channels were the dominant pathway for consumers in 12 of 18 seasonal food webs, with the remaining 6 being more reliant on detrital energy channels. There were contrasting spatiotemporal trends between river networks. Shark River Slough food webs showed a clearer pattern of greener marsh food webs upstream switching to browner food webs more heavily reliant on mangrove detritus downstream. In contrast, Taylor Slough food webs showed the opposite pattern of browner marsh food webs upstream switching to greener food webs downriver and in marine seagrass habitats. Seasonal switching of the dominant energy channel was less common than expected, with 2 of 9 food webs shifting from green to brown dominance between the dry and wet season. Seasonal shifts disrupted spatial gradients in energy channel use, but the seasonal dynamics quantified in this single year study require further contextualization. Our findings provide a short but dynamic view of energy pathways in aquatic communities across the Everglades, but continued research will allow us to better predict how species, food webs, and ecological networks may respond to environmental drivers under future global change.

## Introduction

The flow of energy through food webs is fundamental to our understanding of ecosystem structure and function [1]. Energy from basal resources typically enters consumer food webs via two distinct but highly-coupled energy channels: fast ‘green’ energy pathways and slow ‘brown’ energy pathways [2,3]. Green energy pathways describe more traditional trophic linkages that include the direct consumption of primary producers and the more rapid incorporation of basal resources into consumer biomass [1]. In aquatic food webs, phytoplankton and epiphytic microalgae (EMA) are good examples of green energy channels, as these basal resources are fast growing primary producers with quick turnover rates, meaning there is less time between production and consumer ingestion relative to organic compounds that must first enter detrital pathways [4]. Unlike green energy channels, brown pathways describe microbial mineralization of detrital nutrients and are known to cycle resources that limit rates of basal autotrophic production [5,6]. Most primary producer biomass is not initially consumed, and instead, much of the organic material fixed in an ecosystem is not incorporated into consumer food webs until after decomposition. In aquatic systems, terrestrial plant material from mangrove leaf litter or sawgrasses as well as difficult to ingest submerged aquatic vegetation are good examples of brown pathway basal resources. This unconsumed organic material enters the microbial loop [7], where bacterial activity modifies amino acids, fatty acids, and other essential biomolecules into labile forms that more traditional consumer food webs can use [8–12]. The quantity and quality of available brown energy channel resources varies across ecosystems [2], but generally brown pathways are known to have longer food chains, driven partly by the molecular complexity of detrital material, leading to a less efficient transfer of energy to consumers [8,13,14]. Hence, even slight shifts in the relative contributions of brown and green energy pathways in consumer diets can have implications for meeting their metabolic demands, which over time could alter structure and function on a scale from individuals to ecosystems.

Coupling between brown and green energy pathways is typically mediated by generalist consumers that occupy higher trophic levels and this coupling contributes to ecosystem stability through weak trophic interactions spread across several taxa and functional groups [1,15,16]. Shifts in brown and green energy pathway contributions to consumers can have consequences for ecosystem function by influencing trophic position, predator-prey interaction strengths, and niche sizes [1,14,17–21]. Further, consumers that rely heavily on either brown or green energy pathways may respond differently to press and pulse disturbances, providing additional need to understand the mechanisms driving energy dynamics in both pathways simultaneously [18,22,23]. Evaluating changes in brown and green energy channel contributions is critical for determining ecosystem function in a changing world. Still, few studies have evaluated spatiotemporal variability in brown and green energy channel contributions to entire food webs across large heterogeneous landscapes, mainly due to the magnitude and cost of the undertaking.

Conducting extensive food web studies that evaluate both energy pathways at the ecosystem scale is logistically challenging and costly as it requires intensive sampling over large spatial areas. Still, spatial coverage is essential because consumer-resource interactions do not happen in isolation and can link food webs across habitats and environmental gradients [8,15,24–26]. There is a growing body of literature that attempts to integrate landscape ecology with food web theory to determine how spatial gradients of environmental conditions, such as temperature, salinity, and hydrology influence food web structure and function at the landscape level [27–32]. Environmental gradients across heterogenous landscapes alter rates of primary production shifting the abundance and distribution of basal resources, which drives consumers to either relocate or adjust their diets to meet metabolic demands [16,33–36]. These gradients affect food web structure and function at a given location and shape consumer reliance on each energy pathway. For example, in high-latitude marine ecosystems, environmental gradients influence species turnover rates and ultimately drive seasonal food web complexity and structure [37]. One of the only long-term studies that compares brown and green energy pathways in an estuarine system with a salinity gradient found that basal resource energy contributions are more variable annually at freshwater sites and that the tidal height throughout the system influences the trophic level of a widely dispersed consumer species [38].

In addition to evaluating food web energy pathways across large spatial gradients that accurately represent ecosystem processes, there is a need to account for temporal variability in food web energy dynamics [39]. Most ecosystems exhibit natural variation across numerous temporal scales (e.g., daily, seasonal, yearly, or decadal), and understanding how food webs respond to environmental drivers and perturbations over these time frames is a pivotal goal of ecology [39,40]. Several studies have indicated that seasonality strongly influences food web energy dynamics [41–44]. Long-term studies over years to decades have also demonstrated temporal shifts in food web structure and function driven by anthropogenic activity, with implications for community composition and consumer-resource interactions [36,45–47]. Accounting for temporal variability in food web studies is even more critical given that anthropogenic activities alter the magnitude, timing, and duration of physical and biological processes that shape ecosystems [16,37,48]. While this study is limited to a single year of data with two distinct seasonal food webs, it serves as the foundation for contextualizing food web energy dynamics on spatiotemporal scales that reflect community structure and function in the Everglades.

In this study, we evaluated multichannel food web energy dynamics in nine spatially extensive but interconnected food webs across an ecosystem and seasonal gradient in two river drainages across the Florida coastal Everglades. We asked, do contributions from green and brown energy pathways vary spatially and seasonally for aquatic food webs across an ecosystem gradient of marsh, riverine, and marine habitats. Our study had three primary objectives. First, quantify the proportional contributions of brown and green energy pathways to whole food webs in the Florida coastal Everglades, comparing contributions across habitats and drainages. Second, we aimed to determine if site-specific contributions from brown and green energy channels varied seasonally, as this system exhibits distinct hydrologic wet and dry seasons known to influence consumer-resource interactions [25,49,50]. Finally, we compared seasonal contributions of site-specific basal resources to elucidate spatial and temporal patterns in food web structure and function in coastal systems with freshwater to marine habitat gradients. We used tri-isotope (δ^13^C, δ^15^N, & δ^34^S) Bayesian mixing models to quantify the proportional amount of energy consumers derived from brown and green basal resources, and our models provide a coarse but functional view of energy flow through the system.

## Materials and methods

### Study sites

**Fig 1:**
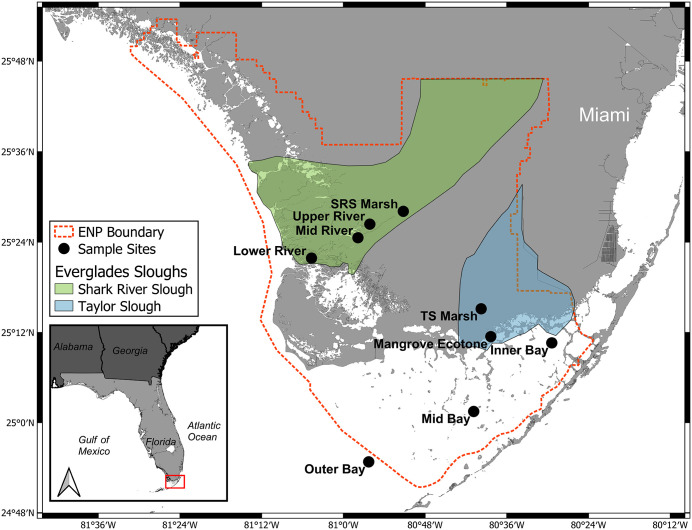
Map of sampling sites. Map of the Florida coastal Everglades with two shaded polygons showing the watersheds of Shark River Slough (green) and Taylor Slough (blue). White points (N = 9) are aquatic food web sampling sites across both river networks. The boundary of Everglades National Park (ENP) is outlined (orange dashed line). All map figures were produced using QGIS (version 3.26.1), an open-source geographic information system software. The resulting maps and data layers are fully compatible with all CC BY 4.0 licenses and can be made available on request.

In southern Florida, United States marsh, riverine, and marine habitats create an interwoven mosaic of aquatic communities unified through sheet flow, that slowly transports freshwater south from Lake Okeechobee to Florida Bay [50,51]. Within this mosaic, we sampled aquatic food webs (N = 9) along two river drainages in Everglades National Park; Shark River Slough (SRS) and Taylor Slough (TS). Food web sampling sites were a subset of those monitored by the Florida Coastal Everglades (FCE) Long-term Ecological Research (LTER) program. These sites were selected to include biological communities across the ecosystem gradient and the ecotones between them (Fig 1). The coastal Everglades exhibits hydrodynamic cycles that create distinct wet and dry seasons annually, but flows have been heavily modified with the magnitude of this hydrologic pulse varying across habitats and river drainage [49,50].

SRS has a drainage area of roughly 1700 km^2^, and begins in freshwater marshes with oligohaline creeks that flow into riverine mangrove and bay ecotones, before eventually flowing into Ponce de Leon Bay and the more saline Gulf of Mexico [52,53]. We sampled four sites along the SRS drainage, ordered here by descending distance from the river delta and increasing levels of salinity: SRS Marsh, Upper River, Mid River, and Lower River. The SRS Marsh site is a freshwater marsh dominated by sawgrass (*Cladium jamaicense*) communities that experience strong seasonal water-level fluctuations. The Upper River is a freshwater site in the Rookery Branch of SRS, representing the ecotone between marsh and riverine communities. The Mid River site is within Tarpon Bay and is a brackish open water bay location with seasonal variability in salinity. The Lower River site is close to the river delta and experiences strong daily fluctuations in salinity due to tidal forces, representing the transition from riverine to marine systems.

TS begins in a freshwater marsh that flows into an oligohaline mangrove ecotone before eventually reaching the shallow seagrass meadows of Florida Bay. [52,54,55]. We sampled five sites along the TS drainage, ordered here from most upriver to the furthest offshore in Florida Bay: TS Marsh, Mangrove Ecotone, and the Inner, Mid, and Outer Bay sites. TS Marsh is a freshwater marsh characterized by seasonal dry downs. The Mangrove Ecotone site is a shallow bay near the river delta, representing the ecotone between riverine and marine habitats. Seagrass meadows dominate the three fully marine sites in Florida Bay. The entire system is oligotrophic, predominantly limited by phosphorus (P), but upstream agricultural inputs and historic reductions in freshwater flow that induced saltwater intrusion led to increased inputs of legacy and marine P to the system, with SRS being more P enriched than TS [52,54,56,57].

### Sample collection

Sample collection occurred primarily during the later stages of the hydrologic dry and wet seasons of 2019 (March-May & October-December, respectively). We collected bulk plant tissue material from multiple organisms to create composite replicates of four basal resource groups from vegetative or algal sources at each site each season, with a few exceptions to fill data gaps that are described in the following paragraphs. The four basal resources used in each food web mixing model were consistent for sites between seasons but varied across sites as expected when sampling the primary producers of different aquatic habitats. For all sites, there were two basal resource representatives from each energy pathway. There were nine types of basal resources used across our study system and in general they can be grouped into algal and vegetative sources that represent green and brown energy pathways respectively. Epiphytic microalgae (EMA), pelagic microalgae (PMA), filamentous green algae (FGA), and periphyton (Peri) were considered green pathway basal resources. These green basal resources consist of living autotrophs, are generally smaller, and were inferred to be more palatable to aquatic consumers. Sawgrass (*Cladium jamaicense*), red mangrove leaf litter detritus (*Rhizophora mangle*: Mang), seagrasses (*Thalassia testudinum, Halodule wrightii, and Syringodium filiforme*), flocculant organic matter (Floc), and red macroalgae from the genus *Bostrychia* (RMA), were considered brown pathway basal resources. These brown resources either consist of non-living organic matter actively being modified by microbial decomposition or were standing stock vegetation that was inferred to enter the detrital pathway due to low palatability for aquatic taxa. All vegetative basal resource samples were composite samples from multiple individuals spaced at least 5m apart. Green pathway basal resources like periphyton and FGA could be collected in this way, but epiphytes and pelagic microalgae had to be collected via other methods.

There were a few exceptions to our bulk tissue basal resource sampling methods described above. Epiphytic microalgae (EMA) were collected by carefully separating epiphytes from red mangrove roots, lily pads, or seagrass blades using a scalpel, but were still collected from multiple macrophytes spaced at least 5m apart. We sampled PMA, representing phytoplankton green energy channels, with two replicate plankton tows at all marine sites in Florida Bay. PMA isotopic values for TS Marsh and all four SRS sites could not be collected. Instead, an average of previously reported phytoplankton isotope values was used to generate source PMA values in our mixing models [58]. PMA source values for SRS food webs were the average of the two closest sites with reported phytoplankton isotope values [58, Table 2], and the TS Marsh site used recently reported isotopic values for microalgae [59]. Similarly, mangrove detrital leaves were not collected at the Outer Bay site. Instead, we used the mean and standard deviation of all red mangrove detritus samples from the other 3 Florida Bay sites (Mangrove Ecotone, Inner Bay, and Mid Bay) to generate mangrove source values for the Outer Bay mixing models. Mangrove leaf litter is consider a brown basal resource as it is not directly ingested by aquatic consumers and instead enters the food web via detrital pathways [60]. Epiphytic microalgae at the Inner Bay site were only collected during the dry season, as was the case for RMA and FGA at the Lower River site. Seagrass source values were generated from multiple seagrass species (turtle grass; *Thalassia testudinum*, manatee grass; *Syringodium filiforme*, or shoal grass; *Halodule wrightii*) depending on the seagrass community structure of the site, but were still generated from bulk tissue replicates of all present species. We considered seagrass species as functionally similar and as a brown basal resource since aquatic consumer species collected in this study do not directly consume seagrasses and much of the seagrass biomass enters the food web as detritus [61]. In addition, 41 of the 983 total samples processed for isotopic analyses were primary producers collected from several sites during the dry season of 2020 to improve basal resource representation during the dry season in our mixing models. To account for lower water levels that limit access, we conducted our dry season sampling slightly earlier for marsh food webs compared to riverine and marine sites.

**Table 2:** Table of basal resource contributions. Comparison of mean seasonal energy contributions from four site-specific basal resources to aquatic consumer communities sampled at nine sites in the coastal Everglades. Mean contributions for each resource are bolded and 95% confidence intervals are reported in parentheses. Shark River Slough food webs (above solid line) are ordered here by descending distance from the river delta and increasing levels of salinity. Taylor Slough food webs (below solid line) are ordered here from most upriver to furthest offshore in Florida Bay.

Site	Basal Resource	Dry Season Mean ± SD (95% CI) Contribution	Wet Season Mean ± SD (95% CI) Contribution

SRS Marsh	Floc	**0.24 ± 0.17 (0.01-0.62)**	**0.18 ± 0.14 (0.01-0.52)**
	Periphyton	**0.10 ± 0.08 (0.00-0.29)**	**0.10 ± 0.09 (0.00-0.32)**
	Phytoplankton	**0.61 ± 0.15 (0.28-0.85)**	**0.69 ± 0.12 (0.40-0.91)**
	Sawgrass	**0.05 ± 0.04 (0.00-0.15)**	**0.03 ± 0.03 (0.00-0.12)**
Upper River	Epiphytes	**0.11 ± 0.09 (0.01-0.33)**	**0.14 ± 0.10 (0.00-0.37)**
	Floc	**0.28 ± 0.12 (0.06-0.51)**	**0.24 ± 0.11 (0.04-0.48)**
	Mangrove	**0.14 ± 0.11 (0.01-0.41)**	**0.19 ± 0.14 (0.00-0.47)**
	Phytoplankton	**0.46 ± 0.12 (0.24-0.72)**	**0.43 ± 0.11 (0.25-0.68)**
Mid River	Epiphytes	**0.11 ± 0.08 (0.00-0.31)**	**0.05 ± 0.05 (0.00-0.20)**
	Floc	**0.12 ± 0.09 (0.00-0.33)**	**0.07 ± 0.08 (0.00-0.28)**
	Mangrove	**0.43 ± 0.08 (0.25-0.57)**	**0.48 ± 0.05 (0.35-0.57)**
	Phytoplankton	**0.34 ± 0.09 (0.15-0.50)**	**0.39 ± 0.09 (0.19-0.52)**
Lower River	Filamentous Green Algae	**0.14 ± 0.08 (0.02-0.32)**	**0.10 ± 0.08 (0.01-0.30)**
	Mangrove	**0.24 ± 0.08 (0.11-0.44)**	**0.69 ± 0.13 (0.43-0.91)**
	Phytoplankton	**0.60 ± 0.11 (0.32-0.76)**	**0.20 ± 0.10 (0.04-0.42)**
	Red Macroalgae	**0.02 ± 0.02 (0.00-0.08)**	**0.01 ± 0.01 (0.00-0.02)**
TS Marsh	Floc	**0.49 ± 0.22 (0.07-0.87)**	**0.52 ± 0.33 (0.02-0.96)**
	Periphyton	**0.25 ± 0.18 (0.01-0.66)**	**0.39 ± 0.33 (0.00-0.93)**
	Phytoplankton	**0.20 ± 0.14 (0.01-0.52)**	**0.05 ± 0.04 (0.00-0.16)**
	Sawgrass	**0.07 ± 0.07 (0.00-0.24)**	**0.03 ± 0.05 (0.00-0.17)**
Mangrove Ecotone	Epiphytes	**0.38 ± 0.16 (0.05-0.68)**	**0.25 ± 0.12 (0.02-0.48)**
	Mangrove	**0.05 ± 0.04 (0.00-0.14)**	**0.16 ± 0.09 (0.00-0.34)**
	SPOM	**0.22 ± 0.16 (0.01-0.61)**	**0.34 ± 0.23 (0.01-0.82)**
	Seagrass	**0.35 ± 0.14 (0.10-0.64)**	**0.26 ± 0.11 (0.06-0.49)**
Inner Bay	Epiphytes	**0.35 ± 0.17 (0.06-0.71)**	**0.33 ± 0.22 (0.03-0.84)**
	Mangrove	**0.18 ± 0.10 (0.02-0.40)**	**0.15 ± 0.12 (0.01-0.41)**
	SPOM	**0.24 ± 0.16 (0.01-0.61)**	**0.31 ± 0.24 (0.01-0.80)**
	Seagrass	**0.23 ± 0.12 (0.02-0.48)**	**0.20 ± 0.14 (0.01-0.53)**
Mid Bay	Epiphytes	**0.48 ± 0.17 (0.09-0.76)**	**0.20 ± 0.11 (0.03-0.45)**
	Mangrove	**0.07 ± 0.04 (0.01-0.16)**	**0.07 ± 0.04 (0.00-0.14)**
	SPOM	**0.21 ± 0.15 (0.01-0.59)**	**0.27 ± 0.18 (0.01-0.62)**
	Seagrass	**0.24 ± 0.10 (0.08-0.47)**	**0.46 ± 0.12 (0.24-0.69)**
Outer Bay	Epiphytes	**0.55 ± 0.26 (0.03-0.91)**	**0.56 ± 0.29 (0.01-0.96)**
	Mangrove	**0.03 ± 0.03 (0.00-0.10)**	**0.02 ± 0.02 (0.00-0.08)**
	SPOM	**0.35 ± 0.27 (0.02-0.92)**	**0.36 ± 0.31 (0.01-0.97)**
	Seagrass	**0.07 ± 0.06 (0.00-0.22)**	**0.06 ± 0.05 (0.00-0.19)**

For consumers, we collected muscle tissue or fin clips from the most abundant species present at a site and intentionally sampled consumers across multiple trophic levels and feeding guilds to have representation of diverse energy pathways (S1 and S2 Figs). The overall consumer community composition of the food web at a given site varied slightly between seasons. A complete list of all consumer isotope values by season (S1 Table) and the contribution of each basal resource to their biomass (S2 Table) is available in the supplementary data repository. Our sampled consumers are not a complete representation of taxonomic diversity, but we consider our sampling method to be effective for quantifying seasonal shifts in brown and green energy pathways across entire food webs because we account for temporal changes in community structure while including variability in resource use from species present at a site during both seasons.

We selected consumer species to represent different functional groups across multiple trophic levels, providing a holistic but coarse representation of aquatic food web structure at each site when possible. Establishing food web structure via functional groups accounts for spatial variability in community composition and reflects conserved functional feeding relationships across sites [62–64]. When possible, the consumers used in each food web mixing model were the same species across sites and seasons, but as expected, community composition varied across the ecosystem habitat gradient and between seasons (S1 and S2 Tables). We collected smaller fishes and invertebrates using seine nets or minnow traps and processed them as composite muscle tissue samples to ensure sufficient sample mass for isotopic analyses. This helps constrain individual variation, thus allowing for an improved portrayal of stable isotope values in lower trophic level species [65–67]. The number of individuals in each composite replicate is reported in the supplemental material (S1 Table). We sampled larger consumers using electrofishing, seine nets, or rod and reel, then collected an anal fin clip prior to release. Using anal fin clips is a non-lethal sampling technique that minimizes risk to consumer fitness but includes different tissue types with variable isotopic turnover rates. We homogenized ray and fin tissues of the clipping, but it should be noted that there could be a temporal mismatch between sample collection and tissue incorporation rates. We attempted to minimize this bias by sampling the later stages of each hydrologic season to give consumers more time to incorporate seasonal prey material. All animal handling and sampling procedures were conducted in accordance with the guidelines of the Florida International University Institutional Animal Care and Use Committee under approved protocol IACUC-23–056-AM01.

### Sample processing

All samples were thoroughly cleaned in deionized (DI) water to remove debris and non-target material. PMA samples were filtered through a glass vacuum and aggregated onto a 0.7µm glass microfiber filter that was previously combusted in a clean muffle furnace at 400°C for 5 hours. All samples were dried in an oven at 55°C for 48 hours, homogenized into a fine powder using a mortar and pestle, and packaged in tin capsules. Calcareous algae, crustaceans, EMA, and PMA required the removal of excess carbonates and sulfates. These samples were decarbonized in silver capsules using 5% hydrochloric acid, rinsed with DI water, dried again, and packaged. Samples were analyzed for carbon, nitrogen, and sulfur stable isotopes (δ^13^C, δ^15^N, and δ^34^S) by the Washington State University Stable Isotope Core Laboratory. All isotope values are expressed in standard δ notation with Vienna PeeDee Belemnite (VPDB), atmospheric nitrogen, and Vienna Canyon Diablo Troilite (VCDT) used as the external reference standards for C, N, and S, respectively. Analytical error, measured as the mean absolute difference of all replicated samples measured across all runs, was 0.4‰ for δ^13^C, 0.5‰ for δ^15^N, and 1.3‰ for δ^34^S.

### Data analysis

Bayesian stable isotope mixing models were run in R [68] using the package MixSIAR [69] to estimate basal resource contributions to entire food webs and individual species during both hydrologic seasons. The basal resource mean and standard deviation values used in each seasonal mixing model for a given site were generated from all composite replicates of that basal resource collected at that site across both seasons. In other words, basal resources were collected during both seasons, but a single mean and standard deviation was generated from all replicates of that resource and used for two mixing models with different seasonal consumer communities. While this method limits our ability to detect seasonal shifts in the isotopic baseline, it does standardize the basal resource pool between seasons and makes our models more conservative by including more variability in basal resource isotopic values used in the mixing models. This helps reduce bias caused by differences in tissue incorporation rates when comparing consumers with varying growth rates and body sizes, but it does not completely remove these challenges when quantifying energy channel contributions to whole food webs on broad spatiotemporal scales. A seasonal comparison of basal resource mean isotopic values is available in the supplementary data repository (S3 Table).

We selected trophic enrichment factors (TEFs) of 1.95 ± 0.3 (mean ± SD) for δ^13^C, 5.1 ± 1 for δ^15^N, and 0.75 ± 0.54 for δ^34^S for all models. The nitrogen fractionation was assumed to represent roughly 1.5 trophic levels [70] and attempts to adjust for fractionation in all consumers while accounting for narrowing discrimination at higher trophic levels [71]. We also chose these TEF values because they enclose consumer isotope values within the mixing polygons (S1 & S2 Figs) and are ecologically relevant to the consumers studied [61,65]. Each model was run using a Markov chain Monte Carlo algorithm comprising of three chains, each with a length of 100000, a burn-in of 50000, and a thinning interval of 50 to ensure model convergence. Our models also included concentration dependency to account for variations in the elemental concentration of basal resources [72]. We nested the species term within the site variable to provide site-level contributions of each basal resource to the entire consumer community directly from the posterior distribution before estimating basal resource contributions to individual species. We summed contributions from pairs of basal resources designated to the same energy pathway to estimate brown and green energy channel contributions at each site. We then used all 3000 of the kept iterations from the mixing model posterior distributions to generate channel-level and resource-specific boxplots for all sites, ensuring that all the variability in the mixing model is expressed on our figures and in our results. Here we report mean energy channel contributions to entire food webs as proportional values from 0–1. Similarly, energy contributions from all four basal resources sum to one for each site each season. All mean values and 95% confidence intervals for contributions of each energy channel or basal resource pool were generated from all 3000 kept iterations of each mixing model output.

## Results

### Brown vs green contributions

**Fig 2:**
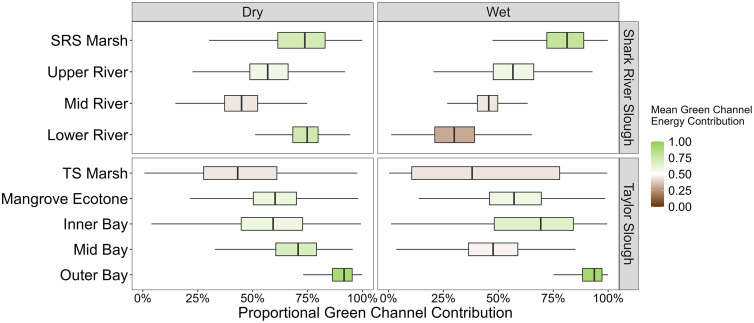
Boxplots of seasonal energy channel contributions. Box plots of the proportional contributions of green energy pathways to aquatic food webs at nine sites during the dry (left panels) and wet (right panels) seasons of 2019 for two coastal drainages, Shark River Slough (top panels) and Taylor Slough (bottom panels). The distribution of the box plots reflects all kept iterations of the posterior distribution for all mixing models with the exclusion of outliers, and fill color is scaled by mean energy pathway reliance.

We found that green energy channels were the dominant pathway (>50% contribution) for most food webs (12 of 18) across both drainages regardless of site and season, but there were notable exceptions with contrasting spatial and seasonal trends between river networks. Spatially, Shark River Slough (SRS) food webs tended to decrease their reliance on green pathways downriver except for the Lower River site during the dry season. In contrast, food webs further downriver and away from shore in Taylor Slough (TS) increased reliance on green energy pathways except for the Mid Bay site during the wet season (Fig 2). The SRS Mid River and TS Marsh sites were the exceptions to green energy pathway dominance and were both slightly more reliant on brown channels with roughly only 45% of the mean energy contributions coming from green basal resources during both seasons for each site (Table 1).

**Table 1:** Seasonal comparison of energy pathway contributions. Comparison of mean seasonal energy contributions from green and brown basal resource pathways to aquatic consumer communities sampled at nine sites across two drainages in the coastal Everglades. Means were generated from all kept iterations of the posterior distributions for each stable isotope mixing model. Shark River Slough food webs (above dashed line) are ordered here by descending distance from the river delta and increasing levels of salinity. Taylor Slough food webs (below dashed line) are ordered here from most upriver to furthest offshore in Florida Bay.

Site	Season	Mean & (95% CI) Green Pathway Contribution	Mean & (95% CI) Brown Pathway Contribution
**SRS Marsh**	**Dry**	**0.29 ± 0.16 (0.06-0.65)**	**0.71 ± 0.16 (0.35-0.94)**
	**Wet**	**0.21 ± 0.13 (0.02-0.54)**	**0.79 ± 0.13 (0.46-0.98)**
**Upper River**	**Dry**	**0.43 ± 0.13 (0.16-0.66)**	**0.57 ± 0.13 (0.34-0.84)**
	**Wet**	**0.43 ± 0.14 (0.14-0.67)**	**0.57 ± 0.14 (0.33-0.86)**
**Mid River**	**Dry**	**0.55 ± 0.11 (0.34-0.77)**	**0.45 ± 0.11 (0.23-0.66)**
	**Wet**	**0.55 ± 0.09 (0.40-0.76)**	**0.45 ± 0.09 (0.24-0.60)**
**Lower River**	**Dry**	**0.27 ± 0.09 (0.12-0.48)**	**0.73 ± 0.09 (0.52-0.88)**
	**Wet**	**0.70 ± 0.13 (0.43-0.92)**	**0.30 ± 0.13 (0.08-0.57)**
**TS Marsh**	**Dry**	**0.55 ± 0.22 (0.14-0.91)**	**0.45 ± 0.22 (0.09-0.86)**
	**Wet**	**0.56 ± 0.34 (0.03-0.98)**	**0.44 ± 0.34 (0.02-0.97)**
**Mangrove Ecotone**	**Dry**	**0.40 ± 0.14 (0.13-0.69)**	**0.60 ± 0.14 (0.31-0.87)**
	**Wet**	**0.42 ± 0.16 (0.09-0.70)**	**0.58 ± 0.16 (0.30-0.91)**
**Inner Bay**	**Dry**	**0.41 ± 0.19 (0.06-0.80)**	**0.59 ± 0.19 (0.20-0.94)**
	**Wet**	**0.35 ± 0.24 (0.03-0.87)**	**0.65 ± 0.24 (0.13-0.97)**
**Mid Bay**	**Dry**	**0.31 ± 0.13 (0.10-0.61)**	**0.69 ± 0.13 (0.39-0.90)**
	**Wet**	**0.53 ± 0.15 (0.26-0.82)**	**0.47 ± 0.15 (0.18-0.74)**
**Outer Bay**	**Dry**	**0.10 ± 0.07 (0.01-0.27)**	**0.90 ± 0.07 (0.73-0.99)**
	**Wet**	**0.08 ± 0.07 (0.00-0.24)**	**0.92 ± 0.07 (0.76-1.00)**

**Fig 3:**
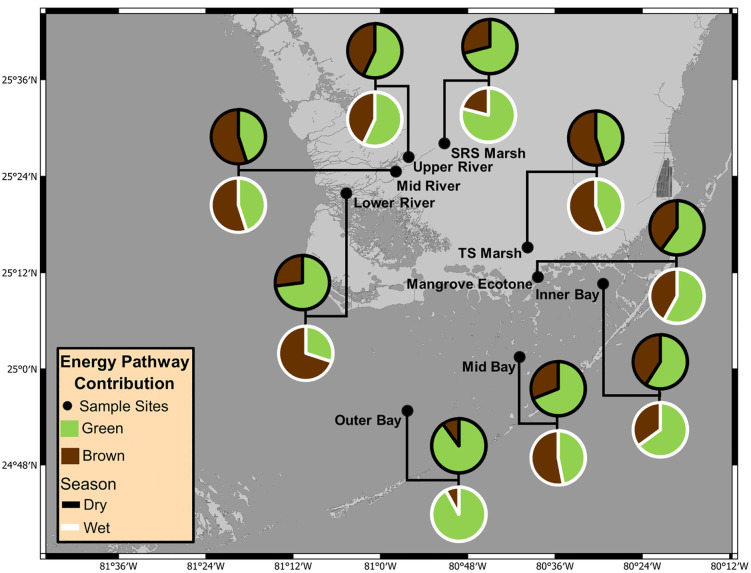
Map of nine food web sampling sites (black dots) across the Florida coastal Everglades with comparative pie charts showing seasonal contributions of green and brown energy pathways to aquatic food webs during the dry (black outline) and wet (white outline) hydrologic season. All map figures were produced using QGIS (version 3.26.1), an open-source geographic information system software. The resulting maps and data layers are fully compatible with all CC BY 4.0 licenses and can be made available on request.

TS Mid Bay and SRS Lower River exhibited the largest seasonal shifts in energy channel contributions and were the only sites to change their dominant energy pathway between seasons. Both sites switched from green-dominated food webs in the dry season to brown-dominated food webs in the wet season (Figs 2–3) but note the seasonal switching disrupted spatial gradients in opposite seasons as mentioned above. Between the dry and wet seasons (Table 1), the mean proportional amount of energy from green pathways decreased from 0.69 to 0.47 at TS Mid Bay and from 0.73 to 0.30 at SRS Lower River. This reflects a 22 and 43% reduction in the overall mean contribution of green energy pathways to the consumer communities at these sites during the wet season. This is roughly 3–4 times greater than the next highest seasonal shifts which were observed at the SRS Marsh and Inner Bay sites. At those sites, the opposite trend occurred with roughly an 8% increase in green pathway contributions between the dry and wet season (Table 1; 0.71 to 0.79 and 0.58 to 0.65 respectively). Energy channel contributions across all other sites remained relatively consistent between seasons regardless of their dominant energy pathway.

### Shark River Slough (SRS) source specific contributions

**Fig 4:**
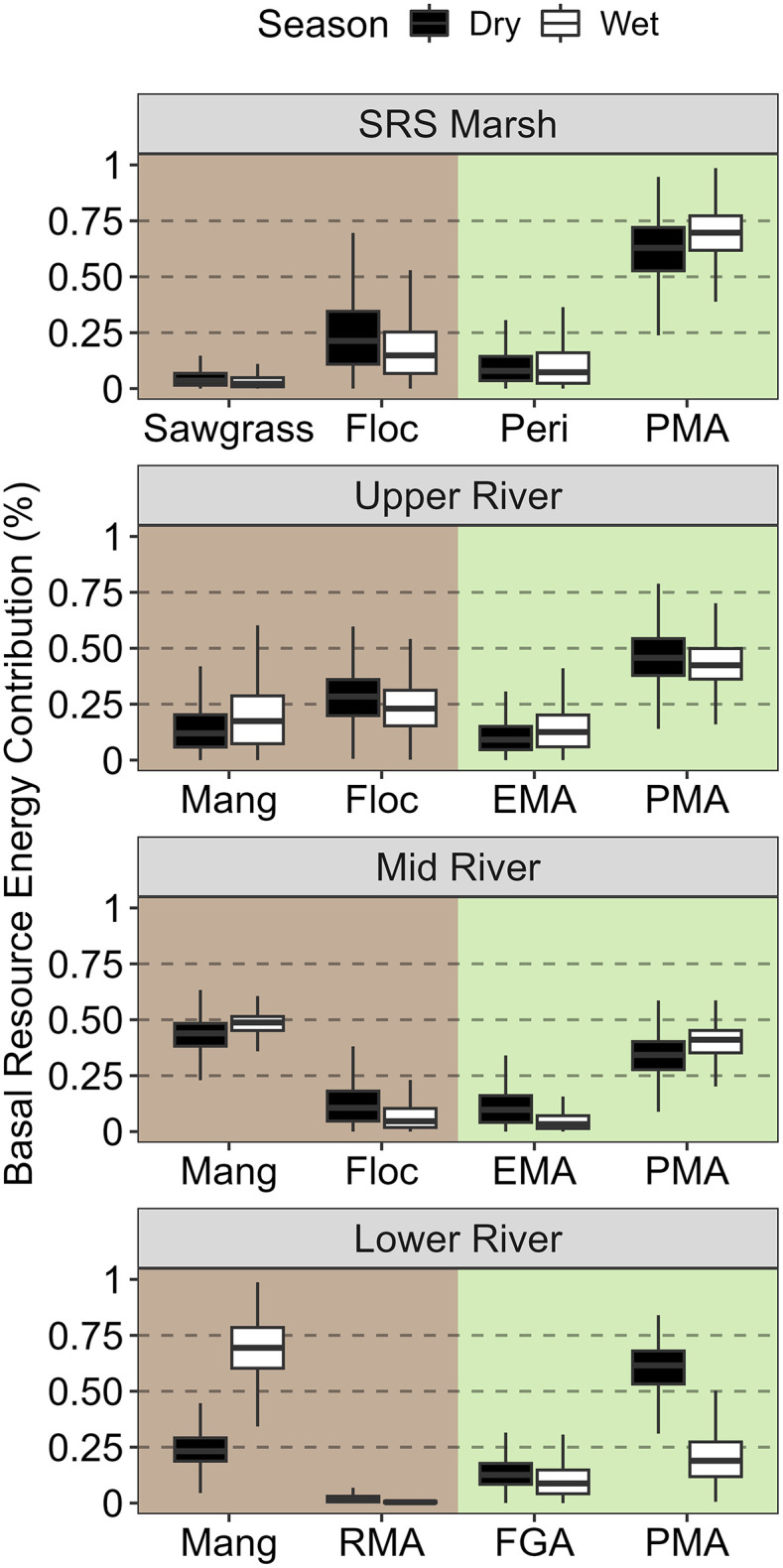
Paired boxplots of Shark River basal resource contributions. Paired box plots comparing seasonal energy contributions of four site-specific basal resource groups during the dry (black) and wet (white) season for coastal food webs sampled along the Shark River Slough transect. Background panel color (brown, left & green, right) corresponds to the energy pathway each basal resource on the x-axis was assigned to. The distribution of the box plots reflects all kept iterations of the posterior distribution for all mixing models with the exclusion of outliers.

Apart from the Lower River site during the dry season, food webs further downriver along the SRS transect were less reliant on green energy channels and site-specific resource use was relatively consistent between seasons (Fig 4). Pelagic microalgae (PMA) contributed most of the energy to consumer food webs at the SRS Marsh site during both seasons, with slightly greater reliance during the wet season (Table 2; 0.61 & 0.69). Flocculant organic matter (floc) contributed more than sawgrass and periphyton to SRS Marsh food webs during both seasons, but none of these resources contributed more than 25% of the proportional energy in either season. PMA also contributed the most proportional energy at the Upper River site but was less dominant contributing roughly 46 and 43% of the energy during the dry and wet season respectively. Floc contributed more energy to the brown channel than mangrove detritus at the Upper River site and epiphytic microalgae (EMA) contributed less than both brown basal resources during both seasons (Fig 4). The Mid River site was slightly more reliant on brown energy pathways overall during both seasons with mangrove detritus contributing more than floc and accounting for 43 and 48% of the proportional energy during the dry and wet season respectively (Table 2). The Mid River food web relied on mangrove detritus more than any other food web in either drainage besides the SRS Lower River site during the wet season. At the Lower River site, we observed a seasonal switch from green to brown energy channel reliance marked with a reduction in contributions from PMA and increased reliance on mangrove detritus between the dry and wet season (Fig 4). The mean proportional contribution of PMA in the Lower River decreased from 0.6 in the dry season to 0.20 in the wet season while mangrove detritus followed the opposite trend and increased from 0.25 to 0.69 (Table 2). Red macroalgae (RMA) and filamentous green algae (FGA) contributions were consistent between seasons in the Lower River (Fig 4).

### Taylor Slough (TS) source specific contributions

**Fig 5:**
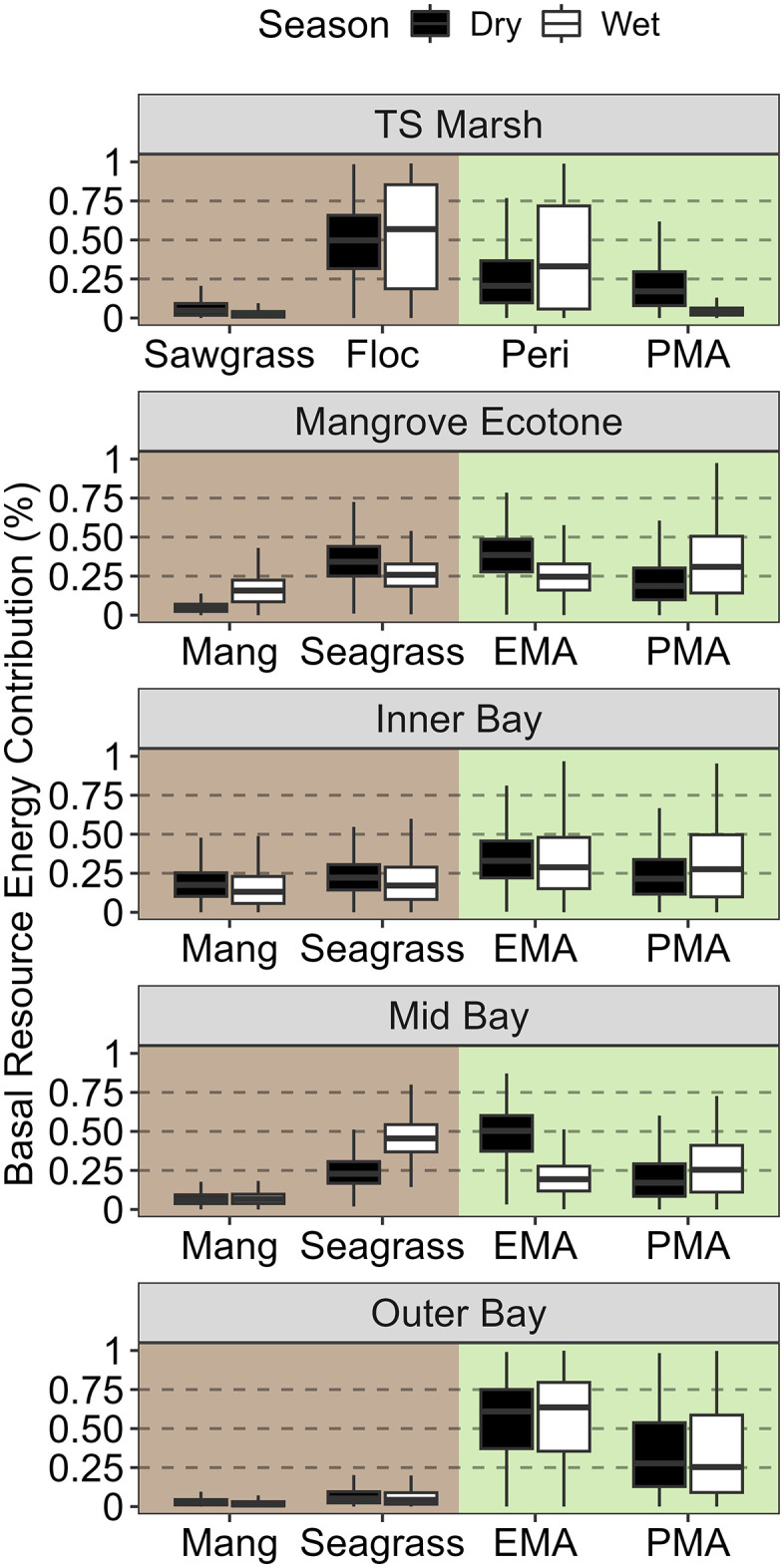
Paired boxplots of Taylor Slough basal resource contributions. Paired box plots comparing seasonal energy contributions of four site-specific basal resource groups during the dry (black) and wet (white) season for coastal food webs sampled along the Taylor Slough transect. Background panel color (brown, left & green, right) corresponds to the energy pathway each basal resource on the x-axis was assigned to. The distribution of the box plots reflects all kept iterations of the posterior distribution for all mixing models with the exclusion of outliers.

Contrary to SRS, food webs along the TS drainage tended to increase reliance on green energy pathways further downriver and away from shore (Fig 2). Brown energy pathways contributed slightly more overall at the TS Marsh site with floc being the most important brown basal resource in both seasons (Table 2; dry: 0.48 and wet: 0.52). Between the dry and wet season at the TS Marsh site, mean contributions from periphyton increased while PMA contributions decreased, and sawgrass contributions remained relatively constant. At the Mangrove Ecotone site, mangrove detritus contributions were minimal, but seagrass, EMA, and PMA roughly contributed equal amounts of energy with greater reliance on seagrasses and epiphytes during the dry season (Table 2, Fig 5). The Inner Bay site was the most consistent between seasons with more uniform contributions from all basal resources. The Inner Bay site was slightly more reliant on green energy pathways with EMA contributing more than PMA during both seasons (Table 2). The Mid Bay site was one of two sites to switch from green to brown energy channel dominance between the dry and wet season. This seasonal switch was marked with a reduction in EMA contributions (0.48 to 0.2) and an increase in seagrass contributions (0.24 to 0.46) while mangrove detritus and PMA contributions remained relatively stable across both seasons (Table 2, Fig 5). The Outer Bay site was dominated by green energy pathways (≥ 90% contributions) during both seasons with EMA consistently contributing slightly more than PMA (Table 2, Fig 5).

## Discussion

Quantifying spatiotemporal trends in energy channel contributions to consumer food webs is critical to a more holistic understanding of energy flow and material cycling through ecosystems [39]. We found that green energy channels were the dominant pathway for 12 out of the 18 seasonal food webs across both coastal drainages, with the remaining 6 seasonal food webs being reliant on detrital energy channels. Shark River Slough food webs show a clearer pattern of greener marsh food webs upstream switching to browner food webs mangrove downstream. In contrast, Taylor Slough food webs showed the opposite pattern of browner marsh food webs upstream switching to greener food webs downstream in seagrass habitats. This contradicts assumptions that brown basal resources would be more important in hyper-oligotrophic systems like the Everglades [8], where production is phosphorus (P) limited [73]. Instead, coastal food webs in the Everglades were largely reliant on green energy pathways, especially during the dry season.

From an energetic perspective, preference of green basal resources by consumers supports the idea that these trophic pathways are shorter and more efficient for meeting metabolic demands [9,13,74]. In aquatic systems, detritus is often allochthonous with higher C:N or C:P ratios (i.e., lower nutritional quality), resulting in preferential omnivory toward standing stock primary producers in green energy channels. This results in faster turnover rates of production in green food webs. [20,75]. In grassland ecosystems, green energy pathways are known to support greater rates of secondary biomass production, and primary producers have higher production-to-assimilation ratios compared to heterotrophic fungi in detrital food webs [76]. For these reasons, the reliance on green energy pathways we observed for consumers in coastal food webs is not necessarily unexpected, as dominant green channels, in conjunction with supportive detrital pathways, may be more capable of supporting complex food webs even in oligotrophic systems like the Everglades [8,77–79]. Despite green pathway prevalence, brown energy channels contributed substantially to most food webs. We provide empirical evidence of spatial variability and seasonal switching of the dominant energy channel across ecological and physiochemical gradients that require further study to contextualize these findings.

There are likely several factors driving spatial and seasonal heterogeneity in brown and green energy channel reliance in the Florida coastal Everglades. Here we propose potential drivers and mechanisms underlying these patterns but recognize the temporal limitation of our single-year study. Additionally, we did not use biomass-weighted abundance in our analysis, as biomass data were not consistently available across all species and sites. Although biomass-weighted models could enhance the accuracy of total energy channel estimates, our approach based on species-level dietary proportions captures key patterns in community structure and resource use. These patterns provide valuable insights into biological processes operating at the ecosystem scale and allow us to explore patterns of resource use driven by species diversity rather than biomass dominance.

Across river drainages we observed contrasting spatial trends, with food webs decreasing reliance on green energy pathways further downriver in Shark River Slough (SRS) while observing increasing reliance on green pathways downriver and away from shore in Taylor Slough (TS). Green food webs tended to be either fully fresh or fully marine with most of the brown dominant and seasonal switching food webs occurring around ecotone sites in both drainages, highlighting the uniqueness and importance of these transitional zones. Hydrology and nutrient availability are likely critical factors driving these spatial patterns [80]. Freshwater flow and P availability is lower in the marsh and riverine sections of TS than in SRS, with total P increasing from freshwater to marine sites in both drainages [80]. The fully marine food webs in Florida Bay were likely green dominant because greater marine P availability leads to reduced nutrient limitation in the phytoplankton and epiphytic microalgae channels of seagrass habitats. Increased reliance on green pathways across Florida Bay could also reflect reduced inputs of terrestrial vegetative sources from brown channels as you move offshore. The increasing proportion of energy consumers derived from green pathways mirrored trends of P availability in TS but was unexpectedly absent across SRS food webs that instead increased reliance on brown pathways towards the coast.

Upstream contributions of legacy phosphorus and stronger freshwater flow in the SRS drainage may prime and transport detrital resources to downstream sites, allowing brown basal resources to be more available to consumers even with increased contributions of marine phosphorus at coastal sites. Cross et al. [81,82] found that experimental enrichment of headwater streams characterized as “detrital based” accelerated detrital processing, resulting in the loss of nearly 50% of in-stream leaf litter. Transport of brown basal resources would explain the overall increased reliance of brown pathways downriver in SRS food webs and possibly explains why marsh habitats were green dominated in SRS Marsh, while brown dominated in TS Marsh. Relationships between food web energy pathway reliance and P availability supports previous work showing the importance of phosphorus in mediating the structure of microbial [81], vegetative [83], and consumer [84] communities in the Everglades, but additional work is needed to compare spatial gradients across river networks. Our findings add to a growing body of literature that suggests that environmental gradients drive spatial variability in food web structure and function in a variety of ecosystems [34,38,85,86].

Previous work in the system has demonstrated seasonal shifts in dissolved organic matter (DOM) composition driven by hydrology, with contributions changing from algal to detrital sources in freshwater marshes, from detrital marsh to detrital mangrove sources in brackish ecotones, and from detrital mangrove to algal marine sources in downstream mangroves between the dry and wet season [87]. We found similar spatial and seasonal trends across energy channels but the unexpected seasonal switching from green to brown energy pathways between the dry and wet season at SRS Lower River and TS Mid Bay may also stem from changes in hydrology with potentially different underlying mechanisms. During the wet season, greater freshwater inputs and flow rates may contribute to the increased transport of previously unavailable carbon into the Lower River where nutrient levels are higher from marine phosphorus contributions. This could spur microbial activity and accelerate the rate at which brown basal resources enter consumer food webs in this habitat relative to upstream sites [88]. Reliance on green energy pathways during the dry season at the Lower River site, could reflect a depleted detrital base or strong influence of marine phosphorus under reduced flow conditions. Contrary to SRS Lower River, the seasonal shift to a brown food web during the wet season at TS Mid Bay may stem from a lack of hydrologic connectivity leading to hypersaline conditions in the middle portion of Florida Bay [89]. High salinities are known to reduce epiphyte availability [90], the primary green energy channel for food webs in this portion of Florida Bay [91,92]. The reduction in epiphyte biomass, and associated increases in detritus quality through seagrass die-offs, may confer a competitive advantage to the microbial loop, potentially driving the shift towards brown energy pathways at TS Mid Bay during the wet season. This shift aligns well with other research in central Florida Bay that observed a similar seasonal switch from green to brown food webs in the wet season [92] and our findings builds on existing work that evaluates how hydrologic connectivity mediates patterns of energy flow through ecosystems [38,93,94]. However, detrital cycling and epiphytic communities in seagrass habitats require additional attention. The relative energetic importance of seagrasses varied between sites in Florida Bay and other studies of seagrass systems in Australia found contradicting levels of importance for seagrasses in marine food webs when using stable isotope compared to fatty acid composition analyses [95]. Greater temporal replication is needed to determine interannual variability and identify the exact mechanisms driving these shifts to further explain why apparent spatial gradients were disrupted in opposites seasons for each drainage despite these two sites following the same general trend of switching from green to brown dominance between the dry and wet season.

Nutrient gradients are intrinsically linked to hydrology on broad spatial scales in the Everglades [96,97] and knowledge of both is essential to understanding of the mechanisms driving spatial and seasonal trends in food web energy dynamics. This work is especially pressing given that Everglades hydrology is changing due to increased freshwater flow from restoration efforts and sea-level rise [98]. Increased freshwater flow from restoration efforts will likely increase the total quantity of phosphorus and other nutrients transported through the Everglades, despite dilution of already oligotrophic water [99,100], and this will likely change the composition of basal resources such as DOM [87]. Nutrients mobilized by increased freshwater flows, alongside increased light availability and warmer temperatures have been shown to increase rates of primary production in aquatic systems [101] and will likely have similar impacts on Everglades food webs. Changes in hydrology will also impact trophic structure and consumer-resource interactions. For example, an ecosystem level analysis of Everglades food web characteristics found that the trophic level of eastern mosquitofish (*Gambusia holbrooki*) and riverine grass shrimp (*Palaemonetes paludosus*) increases under wetter, non-drought conditions [102]. We hypothesize that Everglades food webs will become more reliant on green energy pathways under future freshwater flow conditions with implications for niche partitioning [17,103] and trophic dynamics [13,102,104]. Consumer species that are better adapted to using green energy pathways might proliferate, affecting the overall community composition and ecosystem carbon budget [13]. Mobile generalist consumers are known to spatially link food webs, couple energy channels, support community stability, and could serve as indicator species of functional change [1,15,39]. To contextualize our findings and evaluate interannual variability, future work should further consider energy contributions from specific basal resources to individual species when additional years of data become available and evaluate how environmental drivers shape shifts in energy channel use. It will be important to continue to evaluate energy flow on broad spatiotemporal scales while also considering fine-scale and species-specific changes to aquatic food webs.

In summary, our study evaluated spatiotemporal variability in proportional contributions from green and brown energy pathways to food webs across an ecosystem gradient. Our results highlight the importance of evaluating food webs across multiple scales as we observed variation in food web function across river systems, habitats, and between seasons at the resource and energy channel level. Our work offers a possible sampling design to compare food webs with different community compositions which is necessary to conduct ‘extensive and exhaustive’ [105] food web studies with a multichannel energy pathway perspective. Our study is a representative snapshot of energy dynamics in nine food webs throughout the Everglades ecosystem but long-term monitoring is essential to contextualize our findings and track variation across time as restoration and press or pulse disturbance events continue to alter Everglades food web structure and function [106]. The Florida Coastal Everglades Long-Term Ecological Research program (FCE-LTER) was pivotal for conducting this study as previous work and long-term data contributed to our interpretations of the underlying mechanisms driving spatiotemporal heterogeneity in coastal food web energy dynamics [107]. The LTER network is an invaluable resource that provides the framework necessary for scientists, policy makers, and society to make knowledgeable decisions on how to properly manage and conserve ecosystems. Still, it remains logistically difficult to conduct food web studies on scales that capture the spatial and temporal complexity of energy flow and carbon fluxes in natural ecosystems. Long-term monitoring and continued food web sampling is needed, given that food web studies are invaluable for anticipating how populations, communities, and ecological networks will respond to environmental dissonance under future global change [16].

## Supporting information

S1 Fig Carbon/Nitrogen biplots. Stable isotope biplots comparing δ^13^C and δ^15^N values of consumer species (points) in several functional groups (shape) during the dry (orange) and wet (blue) hydrologic season for nine aquatic food webs in the Everglades. Points are averages from all replicate samples of that given consumer. Black dots with dotted lines are adjusted mean source isotopic values with standard deviations used in the mixing models.
(TIFF)

S2 Fig Carbon/Sulfur biplots. Stable isotope biplots comparing δ^13^C and δ^34^S values of consumer species (points) in several functional groups (shape) during the dry (orange triangles) and wet (blue squares) season for nine aquatic food webs in the Everglades. Points are averages from all replicate samples of that given consumer. Black dots with dotted lines are mean source values with standard deviations used in the mixing models.
(TIFF)

S1 Table Table of consumer lengths, mean and standard deviation of isotope values, and number of individuals per composite sample. (CSV)

S2 Table Table of source-specific consumer resource use. (XLSX)

S3 Table Table of seasonal basal isotope values. (XLSX)
