# Supplementary figures and images for: Variation in food web reliance on green and brown energy pathways across ecosystem gradients
Source: PLoS One. 2026 Feb 4;21(2):e0336521. doi: 10.1371/journal.pone.0336521 (PMC12871965; doi:10.1371/journal.pone.0336521)

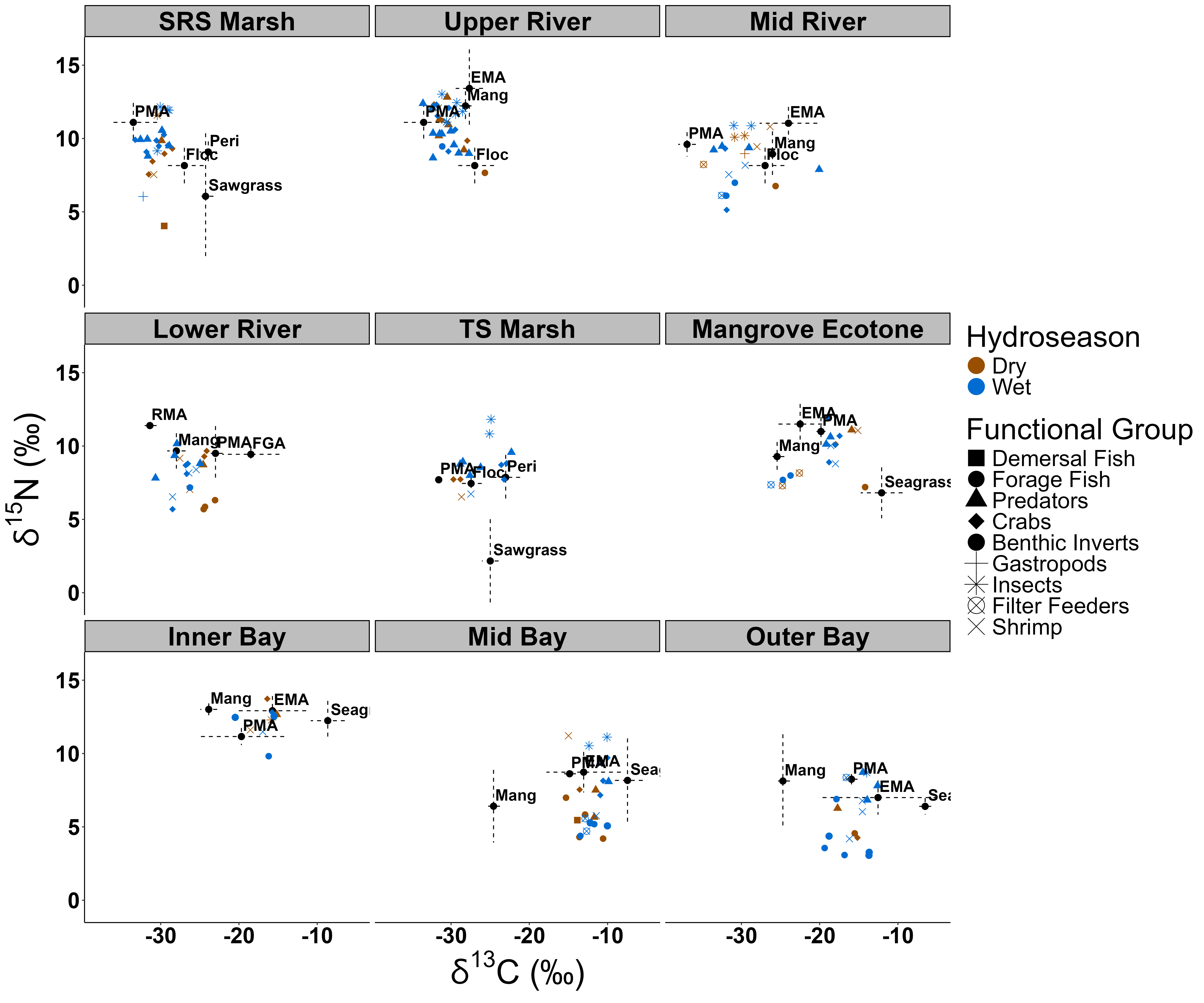

Supplement: S1 Fig — Stable isotope biplots comparing δ13C and δ15N values of consumer species (points) in several functional groups (shape) during the dry (orange) and wet (blue) hydrologic season for nine aquatic food webs in the Everglades. Points are averages from all replicate samples of that given consumer. Black dots with dotted lines are adjusted mean source isotopic values with standard deviations used in the mixing models. (TIFF) [file pone.0336521.s001.tiff]

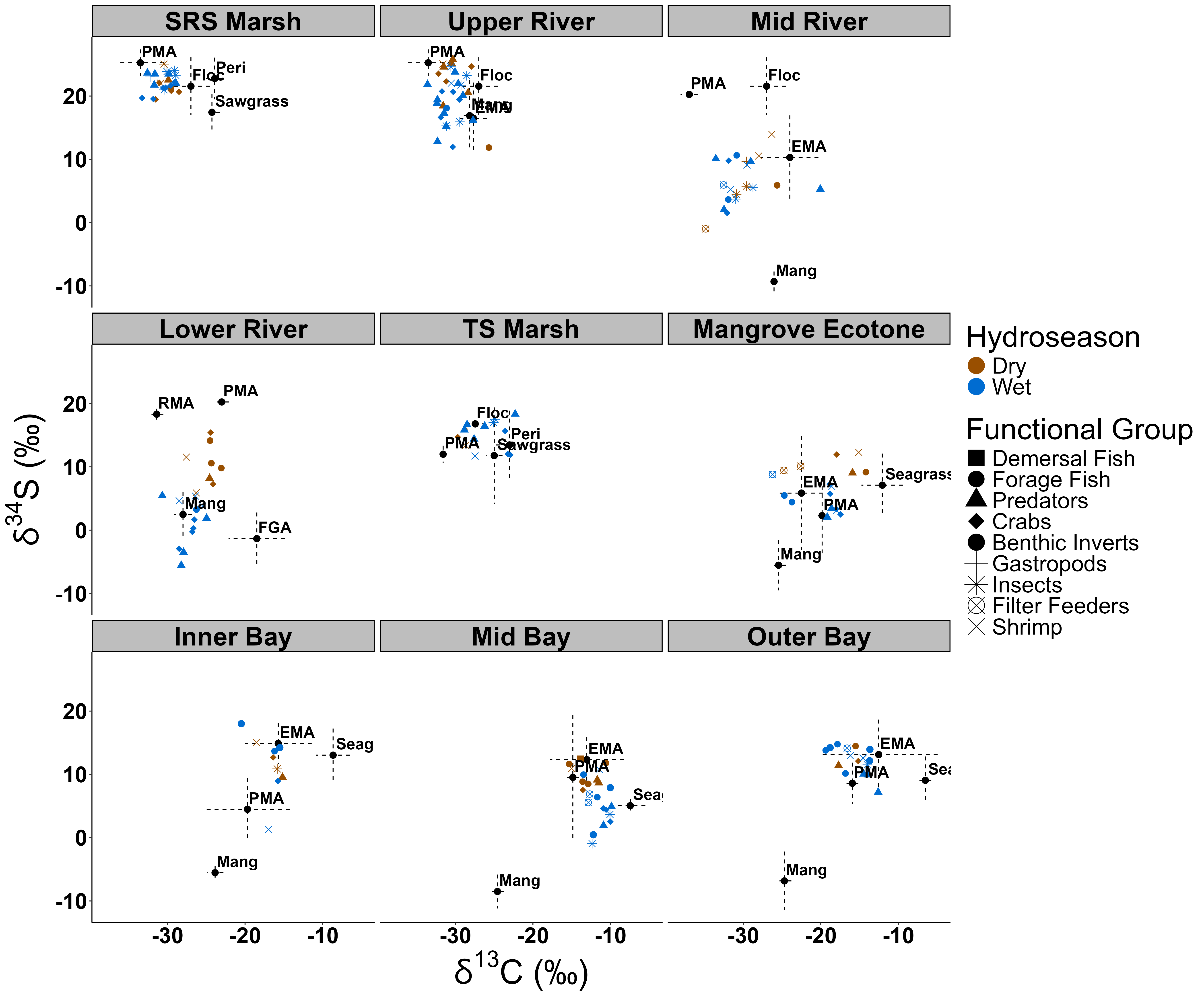

Supplement: S2 Fig — Stable isotope biplots comparing δ13C and δ34S values of consumer species (points) in several functional groups (shape) during the dry (orange triangles) and wet (blue squares) season for nine aquatic food webs in the Everglades. Points are averages from all replicate samples of that given consumer. Black dots with dotted lines are mean source values with standard deviations used in the mixing models. (TIFF) [file pone.0336521.s002.tiff]
